# Virus–Host Interactions and the ARTD/PARP Family of Enzymes

**DOI:** 10.1371/journal.ppat.1005453

**Published:** 2016-03-24

**Authors:** Chad V. Kuny, Christopher S. Sullivan

**Affiliations:** Center for Infectious Disease and Center for Synthetic and Systems Biology, Department of Molecular Biosciences, Institute for Cellular and Molecular Biology, The University of Texas at Austin, Austin, Texas, United States of America; University of Michigan Medical School, UNITED STATES

## Overview

The Poly ADP-Ribose Polymerases (PARPs), also called Diphtheria toxin-like ADP-Ribosyltransferases (ARTDs), catalyze the transfer of ADP-Ribose from nicotinamide adenine dinucleotide (NAD+) to targeted proteins. The activity of these enzymes is generally correlated to cellular stress responses, including oxidative stress, DNA repair, and pathogen infection. Several lines of evidence, including an association with the interferon response, accelerated evolution, and the regulation of viral and antiviral defense transcripts, converge to implicate a widespread involvement of ARTDs/PARPs and ADP-Ribosylation in the mammalian antiviral response. Here, we provide a brief overview of this emerging subfield of virus–host interactions.

## Question 1: What Is an ARTD/PARP?

Poly ADP-Ribose Polymerases (PARPs) are a family of enzymes that transfer one or more ADP-Ribose groups to target proteins, using NAD+ as a substrate. Humans encode 17 PARPs, though the majority of these enzymes are unable to catalyze Poly ADP-Ribosylation (PARylation). Rather, most PARPs transfer a single ADP-Ribose group to target proteins (Mono ADP-Ribosylation or MARylation) [1,2]. The lack of true Poly ADP-Ribose Polymerase activity, as identified through sequence features and biochemical activity, has prompted a nomenclature shift from “PARPs” to the more accurate Diphtheria toxin-like ADP-Ribosyltransferases (ARTDs) (Table 1) [3]. As the name indicates, ARTDs are evolutionarily conserved in organisms from bacteria to humans. In particular, ARTDs and ADP-Ribosylation have strong connections to host–pathogen interactions. While this review will focus on the known and potential contribution of ARTDs to viral infection, it is important to note that several species of bacteria are known to encode ADP-ribosyltransferases that contribute to pathogenesis.

**Table 1:** ARTD/PARP nomenclature.

**ARTD Name**	**PARP Name**	**Other Names**	**Catalytic Activity**
ARTD1	PARP1		PARylation
ARTD2	PARP2		PARylation
ARTD3	PARP3		MARylation
ARTD4	PARP4	vaultPARP	PARylation*
ARTD5	PARP5a	Tankyrase-1	PARylation
ARTD6	PARP5b	Tankyrase-2	PARylation
ARTD7	PARP15	BAL3	MARylation
ARTD8	PARP14	BAL2	MARylation
ARTD9	PARP9	BAL1	Inactive
ARTD10	PARP10		MARylation
ARTD11	PARP11		MARylation*
ARTD12	PARP12	ZC3HDC1	MARylation
ARTD13	PARP13	ZAP, ZC3HAV1	Inactive
ARTD14	PARP7	TIPARP	MARylation
ARTD15	PARP16		MARylation
ARTD16	PARP8		MARylation*
ARTD17	PARP6		MARylation*

The human-encoded ARTDs are listed with their various aliases [3]. Catalytic activity is listed, with asterisks (*) denoting predicted catalytic activity that has not been demonstrated in vitro [1,5].

Similar to other posttranslational modifications, ADP-Ribosylation can exert a wide range of effects on modified proteins, ranging from modification of enzymatic activity to facilitating the ubiquitination and subsequent degradation of targeted proteins [2]. PARylation can also facilitate protein–protein interactions because the heterogeneous and often branched modification provides numerous binding sites for proteins containing WWE, PAR-binding motif (PBM), PAR-binding zinc finger (PBZ), or Macro domains. MARylation, by contrast, is only bound by Macro domain-containing proteins [1,2,4]. ADP-Ribosylation is a reversible modification, and removal of ADP-Ribose is performed by ADP-Ribosylhydrolases (ARH) and the multiple isoforms of the Poly ADP-Ribose Glycohydrolase (PARG) gene. ARH3 and PARG cleave PAR chains to the last ADP-Ribose (MAR), and this last ADP-Ribose group can be removed by certain enzymes that contain a Macro domain (MacroD1 and MacroD2 in humans) or ARH1 [4].

## Question 2: What Are the Cellular Functions of ARTDs/PARPs?

ARTD activity is correlated with cellular stress. The first described and best studied ARTD is ARTD1 (PARP1), which participates in DNA repair. ARTD1 and the related ARTD2 (PARP2) bind to damaged DNA, leading to the PARylation of the ARTDs themselves as well as histones and other nearby proteins. The highly charged PAR polymer then serves as a scaffold to recruit DNA repair enzymes to the site of the lesion. During times of extreme DNA damage, extensive PARylation can deplete NAD+ levels in the cell. In the absence of this important metabolite, ATP production and cellular metabolism is inhibited, which can result in cell death via necrosis [2]. Cellular metabolism is also altered by ARTD8 (PARP14), which promotes the Warburg effect in cancerous cells through regulation of JNK1 [6]. While it is unclear if this regulation is mediated through the enzymatic activity of ARTD8, it seems likely that ARTDs can manipulate cellular metabolism through both the targeting of specific substrates and the consumption of key metabolites [6,7].

**Fig 1:**
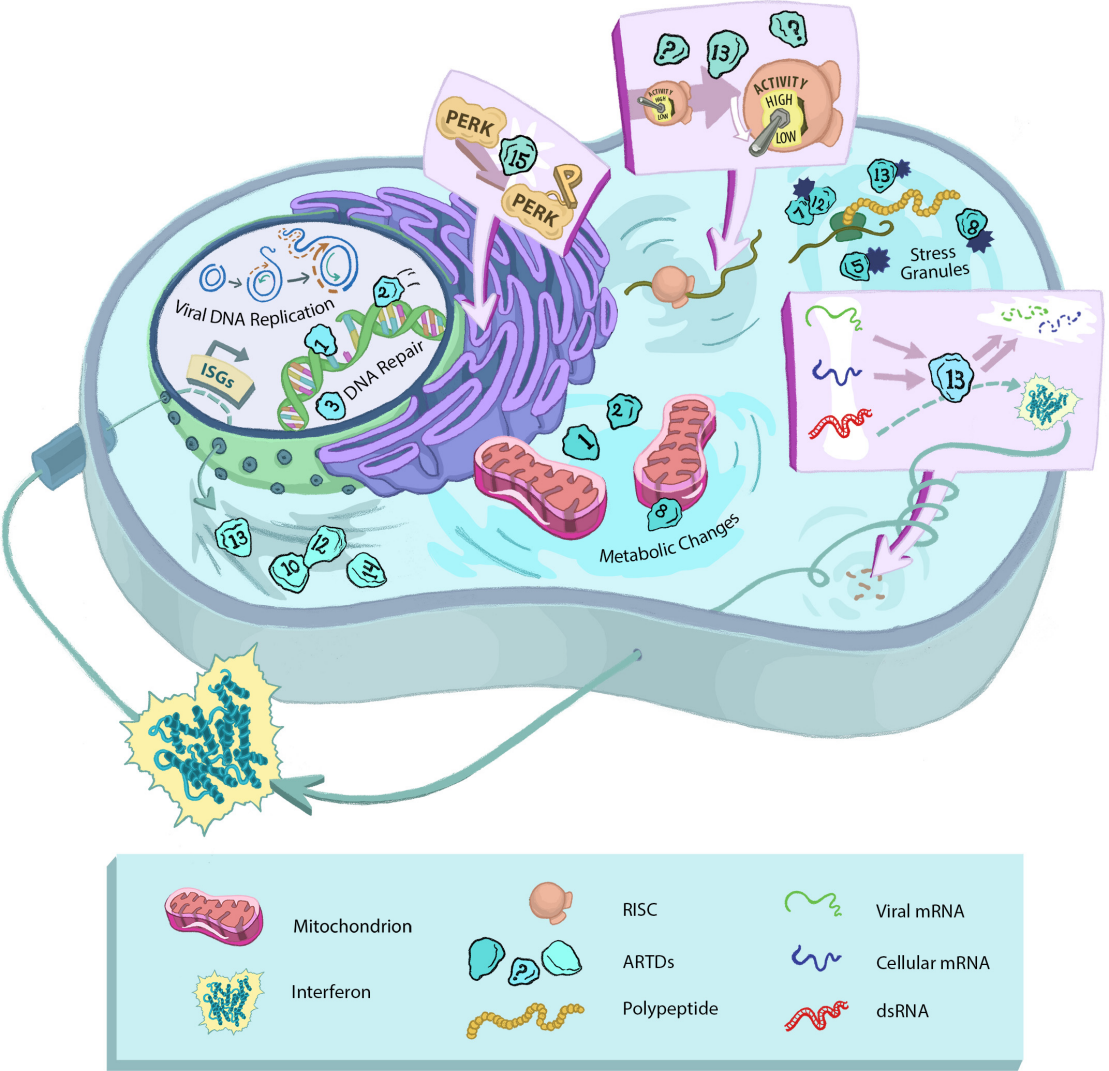
ARTDs/PARPs regulate cellular processes that viruses manipulate. ARTDs are involved in the regulation of multiple cellular stress responses. Virus infection is inherently stressful for the cell and often induces cellular stress responses in the course of replication. Stress-related pathways that are known to overlap between virus infection and ARTD activity are highlighted above. We have emphasized ARTD15’s role in PERK activation [10] and ARTD13’s ability to regulate RNA transcripts through direct (facilitating degradation of RNA [11,12]) and indirect (contributing to RISC inactivation [9]) mechanisms, while contributing to interferon production in response to molecular signatures of viral RNA [9,13]. We also indicate that multiple members of the ARTD family are interferon-stimulated genes (ISGs) [14,15], localize to stress granules [8], and contribute to DNA repair and overall metabolic changes in the cell [2,6,7].

Pioneering work by Chang and colleagues has expanded the view of the ARTD/PARP family beyond their well-established nuclear function in DNA repair to cytoplasmic functions. Systematic characterization studies of the ARTD family have implicated multiple cytoplasmic ARTDs in the regulation of stress responses [1,5]. Varied stressors, including oxidative stress and nutrient deprivation, can lead to translational inhibition and formation of cytoplasmic stress granules around sites of stalled translation. Stress granules contain multiple ARTDs (ARTDs 5, 7, 8, 12, and 13) as well as numerous other proteins. Several proteins in stress granules are ADP-Ribosylated, including the ARTDs themselves, G3BP1, and the Argonaute proteins as part of the RNA-induced silencing complex (RISC) [8]. Although the functional relevance of ADP-Ribosylation in stress granules remains incompletely defined, PARylation of the Argonaute proteins in both stress granules and the free cytoplasm is associated with inactivation of RISC/RNA interference (RNAi) [8,9]. Additionally, ARTD15 (PARP16) has been found to regulate the unfolded protein response through the ADP-Ribosylation of PERK and IREα, which correlates with inhibition of translation [10]. In sum, ARTDs have been implicated in the regulation of multiple cellular stress responses (for a more comprehensive exploration of the cellular activities of ARTDs, see the June 2015 special issue of Molecular Cell). From a virological standpoint, the involvement of ARTDs in these processes is provocative, since each of these responses can be altered during virus infection (Fig 1).

## Question 3: Are ARTDs/PARPs Part of the Mammalian Antiviral Response?

Antiviral activity in the ARTD family was first discovered in the laboratory of Steven Goff, who found that ARTD13, also called ZAP or PARP13, can specifically bind to retroviral RNA, leading to its degradation [11]. This finding was then expanded to include other virus families [16]. ARTD13 can also inhibit endogenous retrotransposition by long interspersed nuclear element (LINE) and Alu elements [14,17]. Furthermore, ARTD13 can directly target cellular transcripts for degradation [12]. ARTD13 targeting of TRAILR4 mRNA promotes TRAIL-mediated apoptosis, which has previously been identified as an antiviral defense mechanism [18]. Therefore, by directly targeting viral and possibly host transcripts for degradation, ARTD13 constitutes part of the antiviral defense.

ARTD13 may also contribute to antiviral defenses by indirectly promoting expression of select transcripts. Cells undergoing oxidative stress or the antiviral response trigger ARTD13-dependent attenuation of RISC-mediated transcript silencing [8,9]. While a direct role of RISC and RNA interference in mammalian antiviral defense remains controversial [19], it is widely accepted that the microRNA (miRNA) component of the RNAi machinery can regulate some pro-death, antiviral, and pro-inflammatory transcripts. Therefore, inhibition of RISC via ARTD activity can conceivably contribute to the innate antiviral defense through the derepression of cytotoxic transcripts [19]. However, as ARTD13 is catalytically-inactive, PARylation-associated inactivation of RISC necessarily requires additional, enzymatically active ARTDs [5,8]. While these additional ARTDs have been identified for oxidative stress-associated inactivation of RISC [8], the relevant ARTDs that attenuate RISC during the antiviral response remain to be determined.

Early clues regarding the identity of antiviral-relevant ARTDs come from comparative genomic studies. This approach can reveal patterns of sequence change that are associated with rapid evolution—a hallmark of pathogen defense proteins. Research from the Malik lab has revealed patterns of ARTD evolution consistent with pathogen response in multiple ARTDs (ARTDs 4, 7, 8, 9, 13) [20,21]. Importantly, some sites of rapid evolution occur in the catalytic PARP domain rather than the RNA-binding domains of ARTDs [20,21]. These studies predict a connection between the antiviral response and the ADP-Ribosyltransferase activity of multiple ARTDs. Consistent with this prediction, several ARTDs (ARTDs 10, 12, 13, and 14) are induced by interferon and can inhibit virus replication, albeit through unknown mechanisms [14, 15]. ARTD13 can also contribute to the production of interferon, facilitating antiviral signaling in response to hallmarks of RNA virus replication [13]. These studies combine to implicate the ARTD family as an underappreciated component of antiviral defense.

## Question 4: Can ARTD/PARP Activity Be Proviral?

Virus infection can manipulate cellular metabolism, induce endoplasmic reticulum (ER) stress, and, with some DNA viruses, invoke or inhibit DNA repair machinery to facilitate genomic replication. Given the central nature of PARylation to the regulation of these processes, it would seem likely that some viruses, at least indirectly, utilize these enzymes to facilitate their replication. A herpes simplex virus 1 (HSV-1) gene product (ICP0) degrades a PARG isoform, suggesting that increased PARylation can be beneficial for viral infection [7]. Also, global inhibition of ARTD activity has been shown to attenuate the replication of a wide variety of viral families, including poxviruses [22], polyomaviruses [23,24], herpesviruses [7], adenoviruses [25], and arteriviruses [26]. Other studies report that ARTD activity affects genomic maintenance or lytic reactivation during the latent cycle of two closely related herpesviruses [27,28] as well as the integration of Hepatitis B and potentially retroviral genomes into host DNA [29,30]. When combined with the antiviral effects of ARTDs mentioned in the previous section, it is clear that a complete understanding of virus–ARTD interactions requires definition beyond a simplistic proviral or antiviral label.

## Question 5: What Is the Future for ARTD/PARP Biology and Viruses?

A growing body of evidence supports a substantial role for ARTDs during virus infection and antiviral defense. ARTDs regulate aspects of cellular biology that viruses routinely manipulate during infection. Multiple ARTDs are interferon-inducible, bear evolutionary signatures consistent with a role in antiviral defense, and have been found to inhibit viral infection. However, despite the mounting evidence for the relevance of ADP-Ribosylation during virus infection, few relevant MARylated or PARylated targets have been described. Recent proteomics approaches have identified PARylated proteins during nonviral stress conditions [31], and similar approaches should prove informative for understanding virus–host interactions. ARTDs, particularly ARTD13, also regulate RNA transcripts through direct and indirect mechanisms [9,11]. Defining these transcripts will also contribute to our understanding of ARTDs in virus–host interactions.

The lack of inhibitors to specific ARTDs handicaps our understanding of individual ARTDs in relation to virus infection. While specific inhibitors have been engineered for ARTD1, which is a target of cancer chemotherapy, studies attempting to target other ARTD family members often utilize the general ARTD inhibitor 3-amidobenzadole (3AB). As a structural mimic of nicotinamide, a byproduct of the ADP-Ribosyltransferase reaction, 3AB likely inhibits numerous ARTDs as well as other ADP-Ribosyltranferases [2]. Future studies utilizing more specific inhibition strategies (e.g., small interfering RNA (siRNA), genetic, or chemical inhibitor approaches) are warranted [32].

While this review has focused on the catalytic activity of ARTDs in regards to viral infection, it should be emphasized that these enzymes are also relevant to viral infection outside of their ADP-Ribosylation activity. ARTD13, which is catalytically inactive, has well-defined antiviral activities [11,16], and other ARTDs likely affect viral replication independently of direct catalysis. Conversely, ADP-Ribosylation is catalyzed by other families of enzymes, including a subset of the sirtuin family [33]. Though sirtuins are outside the scope of this review, we note that these sirtuins possess activities related to infection and pathogen defense [34].

Many questions remain regarding the interplay between viruses and ARTDs. While DNA damage is clearly a trigger for nuclear ARTD activity [2], it remains unclear what cues cytosolic ARTDs during virus infection. Furthermore, it is unknown which ARTD-regulated protein and transcript targets are most relevant to the antiviral response. Whether catalyzed by ARTDs, sirtuins, or other ribosyltransferases, it is likely that ADP-Ribosylation will have a breadth of effects similar to other posttranslational modifications. Therefore, defining the relevant contexts and consequences of MARylation or PARylation of individual targets is imperative to understanding the biology of ARTDs. The interplay between ARTDs and viruses is an underappreciated aspect of virology, and it has the potential to reveal new insight to cellular biology and virology while identifying new therapeutic targets.
